# 恶性胸膜间皮瘤免疫检查点抑制剂的研究进展

**DOI:** 10.3779/j.issn.1009-3419.2021.102.18

**Published:** 2021-06-20

**Authors:** 亚茹 黄, 庆威 孟

**Affiliations:** 150000 哈尔滨，哈尔滨医科大学附属肿瘤医院 Department of Medical Oncology, Harbin Medical University Cancer Hospital, Harbin 150000, China

**Keywords:** 恶性胸膜间皮瘤, 免疫治疗, 免疫检查点抑制剂, 肿瘤异质性, 生物标志物, Malignant pleural mesothelioma, Immunotherapy, Immune checkpoint inhibitors, Tumor heterogeneity, Biomarkers

## Abstract

恶性胸膜间皮瘤（malignant pleural mesothelioma, MPM）是一种侵袭性强、生存率低且缺乏有效治疗选择的恶性肿瘤。培美曲塞联合顺铂作为晚期MPM唯一的一线治疗方案长达20年之久。长期以来，免疫疗法被认为是MPM的一种潜在治疗方法，主要包括免疫检查点抑制剂（immune checkpoint inhibitors, ICIs）、免疫毒素治疗、抗癌疫苗、过继性细胞治疗等。本篇综述重点总结了MPM中ICIs的研究进展，初步分析了MPM肿瘤异质性对ICIs治疗的影响，描述了以生物标志物为导向的免疫治疗是实现MPM个体化治疗的新愿景。

## Abstract (en)

Malignant pleural mesothelioma (MPM) is a malignant tumor with strong invasiveness, low survival rate and lack of effective treatment options. As the only first-line treatment plan for the advanced MPM, combination of pemetrexed and cisplatin chemotherapy have been existing since the last 20 years. Immunotherapy has long been considered as a potential treatment plan for MPM, mainly including immune checkpoint inhibitors (ICIs), immunotoxin therapy, anti-cancer vaccine and adoptive T-cell therapy. This review focuses on summarizing the current research status of immune checkpoint inhibitors in MPM, discusses the effect of tumor heterogeneity on ICIs treatment, and describes that the biomarker-oriented immunotherapy is a new vision for the realization of individualized treatment of MPM.

恶性胸膜间皮瘤（malignant pleural mesothelioma, MPM）起源于胸膜间皮细胞，是一种侵袭性极强且极少被治愈的罕见癌症，占所有癌症病例的0.3%^[1]^。石棉暴露是80%患者的致病因素^[2]^。近年来，免疫治疗中免疫检查点抑制剂（immune checkpoint inhibitors, ICIs）已经很好地改善了多种实体瘤预后^[3]^，ICIs在MPM中也可能具有良好的抗肿瘤作用。

ICIs治疗间皮瘤的早期试验报告了部分患者的临床反应，但是单药ICIs的疗效在MPM中受到限制^[4]^，可预测MPM免疫治疗疗效的生物标志物仍不确定。在这篇综述中，我们重点总结了MPM中ICIs的相应临床研究的最新进展，探讨了生物标志物程序性死亡受体配体1（programmed cell death ligand 1, PD-L1）在ICIs治疗中的意义。

## 1 MPM的组织学异质性

MPM的组织学亚型是治疗决策和预后评估的主要指标，根据肿瘤细胞的形态将其分为3种亚型：上皮样、双相（或混合）和肉瘤样，中位生存期（median overall survival, mOS）分别为19个月、12个月和4个月，其中上皮样与最佳预后和最佳化疗反应相关，肉瘤样预后最差^[5]^。不同组织学亚型表达的免疫检查点不同，肉瘤样MPM中PD-L1表达明显高于其他组^[6]^，上皮样亚型中细胞毒性T淋巴细胞相关蛋白4（cytotoxic T lymphocyte-associated antigen 4, CTLA-4）表达更高^[7]^，T细胞活化的含V区免疫球蛋白抑制物（V-domain Ig suppressor of T-cell activation, VISTA）在上皮样MPM中过度表达^[8]^。有研究^[9]^表明，双相MPM细胞对可协同提高MPM铂类化学疗法的蛋白酶体抑制剂硼替佐米更为敏感。MPM组织分子的异质性有助于开发个性化疗法，尤其是免疫疗法和靶向疗法。

## 2 MPM的肿瘤微环境异质性

肿瘤内和肿瘤间异质性相关的研究大多数都指出了微环境（特别是免疫细胞）的强大作用。MPM微环境的免疫抑制性表现为细胞毒性淋巴细胞（cytotoxic T lymphocyte, CTL）数量少，T细胞上共抑制受体如程序性死亡受体1（programmed cell death 1, PD-1）和T细胞免疫球蛋白黏蛋白-3（T cell immunoglobulin mucin-3, TIM-3）上调，免疫抑制性细胞如髓系来源抑制细胞（myeloid-derived suppressor cells, MDSCs）和调节性T细胞（regulatory T cells, Tregs）增多^[10]^。相关研究^[11, 12]^分析表明肉瘤样MPM中M2巨噬细胞和CD8^+^ T淋巴细胞以及单核细胞和成纤维细胞的水平升高，上皮样MPM中自然杀伤细胞和CD4^+^ T淋巴细胞以及CD20^+^ B淋巴细胞比重大。MPM中上皮样和肉瘤样成分的比例与免疫反应相关，可预测预后和抗肿瘤药物的敏感性^[13]^。

在一项研究^[11]^中，当肿瘤被CD8^+^肿瘤浸润淋巴细胞（tumor infiltrating lymphocytes, TILs）和辅助性T细胞（helper T cell, Th）高度浸润时，MPM患者的生存期更长，相反，也有研究^[12, 14]^表明较高CD8^+^ TIL浸润的MPM患者化疗反应更低、预后更差，可能与CD8^+^ TIL中较高的PD-L1表达相关；M2巨噬细胞比例高是上皮样MPM的阴性预后因素^[15]^。

组织学和微环境异质性之间的相互作用是MPM治疗的主要挑战，也可能是MPM目前治疗机会有限的主要原因之一。

## 3 免疫检查点抑制剂

目前ICIs靶向的两条主要途径是CTLA-4/B7和PD-1/PD-L1轴^[2]^，临床上经常使用的ICIs包括CTLA-4抑制剂Ipilimumab和Tremelimumab，PD-1抑制剂Nivolumab和Pembrolizumab以及PD-L1抑制剂Durvalumab、Avelumab和Atezolizumab。

部分ICIs已经应用在MPM临床治疗中。根据MERIT试验结果，日本于2018年批准了Nivolumab作为间皮瘤的挽救疗法。Ⅲ期临床试验Checkmate 743研究首次证明与化疗相比，Nivolumab联合Ipilimumab的一线治疗可为晚期MPM患者提供长期OS获益^[16]^，据此，美国食品药品监督管理局（Food and Drug Administration, FDA）批准Nivolumab联合Ipilimumab作为晚期MPM的初始疗法。

### 3.1 MPM中ICIs作为挽救性治疗的临床试验

MPM患者标准一线治疗方案的mOS只有约12.1个月^[17]^，进展后的二线治疗疗效更差强人意。因此迫切需要新的挽救疗法。ICIs作为挽救性治疗的试验结果（表 1）为复发或进展的MPM患者带来了新曙光。

**表 1:** MPM中ICIs挽救性治疗的临床试验 Salvage therapy checkpoint inhibitor trials in MPM

Study	Drugs	Phase	*n*	ORR（%）	mPFS (mon)	mOS (mon)	Identifier
KEYNOTE-028^[18]^	K	Ib	25	20.0	5.4	18.0	NCT02054806
NivoMes^[19]^	O	Ⅱ	34	24.0	2.6	11.8	NCT02497508
MERIT^[20]^	O	Ⅱ	34	29.4	6.1	17.3	JapicCTI-163247
JAVELIN^[21]^	B	Ib	53	9.0	4.1	10.7	NCT01772004
PROMISE-meso^[4]^	K *vs* C	Ⅲ	144	K: 22.0; C: 6.0	K: 2.5; C: 3.4	K: 10.7; C: 11.7	NCT02991482
CONFIRM^[22]^	O *vs* P	Ⅲ	332	NR	O: 3.0; P: 1.8	O: 9.2；P: 6.6	NCT03063450
MESOT-TREM-2008^[23]^	Treme	Ⅱ	29	7.0	6.2	10.7	NCT01649024
MESOT-TREM-2012^[24]^	Treme	Ⅱ	29	13.8	6.2	11.3	NCT01655888
DETERMINE^[25]^	Treme *vs* P	IIb	571	Treme: 4.5; P: 1.1	Treme: 2.8; P: 2.7	Treme: 7.7; P: 7.3	NCT01843374
MAPS-2^[27]^	O *vs* O+Y	Ⅱ	125	O: 19.0; O+Y: 28.0	O: 4.0; O+Y: 5.6	O: 11.9; O+Y: 15.9	NCT02716272
INITIATE^[28]^	O+Y	Ⅱ	34	29.0	6.2	12.7-NR	NCT03048474
NIBIT-MESO-1^[29]^	I+Treme	Ⅱ	40	28.0	5.7	16.6	NCT02588131
O: Nivolumab; K: Pembrolizumab; T: Atezolizumab; B: Avelumab; I: Durvalumab; Y: Ipilimumab; Treme: Tremelimumab; P: placebo; C: chemo (Gemcitabine or Vinorelbine); NR: not reached; ORR: overall response rate; mPFS: median progression-free survival; mOS: median overall survival.							

#### 3.1.1 PD-1/PD-L1抑制剂单药临床试验

KEYNOTE -028研究首次对Pembrolizumab单药治疗进行了评估，该研究纳入25例PD-L1表达阳性（TPS≥1%）的患者，疗效数据显示客观缓解率（objective response rate, ORR）为20%，疾病控制率（disease control rate, DCR）为72%，mOS和中位无进展生存期（median progression-free survival, mPFS）分别为18个月和5.4个月，结果令人鼓舞^[18]^。然而，同期的NivoMes、MERIT、JAVELIN研究结果并不亮眼，此3项研究均未将PD-L1表达纳入患者筛选标准。NivoMes试验^[19]^表明PD-L1表达与ICIs治疗反应率之间无明显相关性；MERIT试验^[20]^表明PD-L1表达阴性（TPS < 1%）的患者也获得了临床益处；JAVELIN研究结果^[21]^显示PD-L1表达不同（阈值为5%），ORR和OS也不同。

Ⅲ期随机试验PROMISE-meso结果令人失望，该研究比较了Pembrolizumab与单药化疗（吉西他滨或长春瑞滨）作为二线治疗的疗效。尽管Pembrolizumab的ORR为22%，化疗组为6%，但Pembrolizumab的mPFS为2.5个月，化疗组为3.4个月，未能显示出PD-1治疗的优越性。但是发现了对Pembrolizumab的长期应答者^[4]^，再次说明了解哪些患者受益于PD-1抑制剂治疗的重要性。

另一项以Nivolumab单药治疗的Ⅲ期CONFIRM研究结果可喜，该研究报告了Nivolumab治疗使上皮样MPM患者死亡风险相对下降29%，Nivolumab同时增加了PFS和OS获益；上皮样亚组中Nivolumab和安慰剂组的mOS分别为9.4个月和6.6个月（HR=0.71, *P*=0.021），上皮样MPM患者OS获益显著；该研究表明PD-L1表达对Nivolumab治疗OS无预测价值，但组织学类型可能有预测价值^[22]^。

#### 3.1.2 CTLA-4抑制剂单药临床试验

至今为止，单药CTLA-4抑制剂的临床研究有3项。最初，Ⅱ期MESOT-TREM-2008^[23]^和MESOT-TREM-2012^[24]^试验显示了有希望的结果，因此开始进行以Tremelimumab单药治疗的随机对照试验DETERMINE，该试验结果显示mPFS和mOS未能显著超越安慰剂组，结果令人失望^[25]^。

#### 3.1.3 联合应用免疫检查点抑制剂的临床试验

Ning等^[26]^研究表明CTLA-4抑制剂可诱导T细胞增殖和产生新的抗肿瘤T细胞反应，PD-1/PD-L1抑制剂可恢复现有的T细胞抗肿瘤功能，鉴于联合应用ICIs有较好的理论基础，几项临床试验研究了MPM中ICIs联合治疗的可能性。

Ⅱ期MAPS-2试验表明单用Nivolumab或联合应用Ipilimumab和Nivolumab都有临床意义，两组患者的DCR分别为40%和52%，ORR分别为19%和28%，mOS分别为11.9个月和15.9个月，但是联合治疗组的药物相关不良事件比例略高；同时该研究报告了由于免疫疗法引起的超进展（hyperprogressive disease, HPD）^[27]^，此结果有待深究。

INITIATE试验中也观察到了Ipilimumab+Nivolumab组合的临床活性，ORR为29%，mPFS为6.2个月，但是联合治疗的毒性更大，有94%的患者发生了不良事件，但大多数副作用易于控制，且未观察到5级毒性^[28]^。

NIBIT-MESO-1试验^[29]^是Durvalumab联合Tremelimumab的研究，ORR为28%，DCR为65%，mOS为16.6个月，17.5%的患者出现了3级-4级与治疗相关的副作用，不良事件较MAPS-2研究少。

### 3.2 MPM中ICIs作为一线治疗的临床试验

为进一步提高MPM患者对ICIs治疗的应答率，将ICIs移至一线治疗，并与不同靶点ICIs组合，或将其与化学疗法相结合，从而更有效的恢复免疫系统活力的临床试验（表 2）值得期待。

**表 2:** MPM中ICIs一线治疗的临床试验 Front-line checkpoint inhibitor trials in MPM

Study	Drugs	Phase	*n*	ORR (%)	mPFS (mon)	mOS (mon)	Identifier
Checkmate 743^[16]^	O+Y vs C/P	Ⅲ	605	O+Y: 40.0; C/P: 43.0	O+Y: 6.8; C/P: 7.2	O+Y: 18.1；C/P: 14.1	NCT02899299
DREAM^[30]^	I+C/P	Ⅱ	54	48.0	6.9	18.4	ACTRN12616001170415
PrE0505^[31]^	I+C/P	Ⅱ	55	56.0	6.7	21.1	NCT02899195
IND-227	K *vs* K+C/P *vs* C/P	Ⅱ	520	Ongoing			NCT02784171
DREAM 3R	I+C/P *vs* C/P	Ⅲ	480	Recruiting			NCT04334759
ETOP BEAT-meso	Bev+C/P *vs* T+Bev+C/P	Ⅲ	320	Recruiting			NCT03762018
C/P: Cisplatin combined with Pemetrexed; Bev: Bevacizumab; MPM: malignant pleural mesothelioma.							

近期，Checkmate 743临床试验报告了Ipilimumab+Nivolumab组和化疗组的mOS分别为18.1个月和14.1个月（HR=0.74, *P*=0.002），其中上皮型和非上皮型患者的mOS基本相当（18.7个月*vs* 18.1个月），因此无论组织学类型如何，较化疗组，双免疫治疗均能给MPM患者带来生存获益且不良反应发生率更低；其中非上皮样患者疗效更高，OS增益有显著差异；PD-L1表达阳性（TPS≥1%）的患者OS获益更大^[16]^。

目前，多项ICIs联合化疗的临床研究正在进行或等待评估：如IND-227研究，将化疗与Pembrolizumab联合，探讨一线治疗中Pembrolizumab的价值；基于Ⅱ期DREAM^[30]^和PrE0505^[31]^试验令人欣喜的结果，启动了DREAM3R研究探讨在基于铂的标准疗法中加入Durvalumab与标准化疗的效果；鉴于最近的研究表明贝伐珠单抗在MPM一线治疗中具有疗效，ETOP BEAT-meso试验比较了贝伐珠单抗+卡铂+培美曲塞三联疗法与贝伐珠单抗+Atezolizumab+卡铂+培美曲塞四联疗法治疗新诊断MPM患者的疗效。

### 3.3 MPM中PD-L1表达及其意义

MPM微环境中多种成分可以表达PD-L1，包括肿瘤细胞和浸润的免疫细胞。一项荟萃分析^[32]^表明PD-L1高表达是MPM患者OS而非PFS的阴性预后因素。肿瘤细胞PD-L1表达与MPM中间质TILs的浸润有关，与肿瘤细胞PD-L1高表达相比，间质TILs中PD-L1高表达与临床治疗反应的相关性更强^[26]^。有研究^[14, 33]^表明肉瘤样MPM侵袭性更强，化疗反应更低，更易受益于免疫疗法，可能与肉瘤样亚型中PD-L1^+^ CD8^+^ TIL数量较多有关。

Checkmate 743研究^[34]^报告了双免疫治疗一定程度上逆转了PD-L1的负面预后作用，提示PD-L1表达可能是双免疫治疗的有效预测因素。Terra等^[35]^研究表明PD-L1表达在治疗过程中可能会发生变化，或许限制了抗PD-L1治疗的疗效。ICIs治疗的敏感性可能与PD-L1表达有关，但是部分临床试验表明PD-L1表达低的患者也可从ICIs治疗中受益。

总之，PD-L1作为MPM治疗的预测性生物标志物一直存在争议。PD-L1表达检测阈值的不同、MPM组织学异质性和微环境异质性以及其他免疫抑制和激活因素（例如TILs、Tregs、炎症、HLA分型和微生物组成成分^[36]^等）限制了PD-L1被用作ICIs治疗MPM的预测性生物标志物。

### 3.4 其他潜在的免疫检查点抑制剂靶点

鉴于MPM的肿瘤异质性，靶向CTLA-4和PD-1/PD-L1轴的抑制剂仅可为部分患者带来长期生存获益，因此与治疗相关的其他共抑制和共刺激分子，例如：VISTA^[8]^、TIM-3^[37]^、淋巴细胞激活基因3（lymphocyte-activation gene 3, LAG-3）^[38]^、T细胞共刺激因子OX40（TNFRSF4）及其配体OX40L（TNFSF4）^[39]^以及诱导型T细胞共刺激物（inducible T-cell costimulatory, ICOS）^[40]^，正在研究中，以期为更多MPM患者带来新的免疫治疗希望。

## 4 总结

晚期MPM的ICIs治疗已经取得了期待已久的进步，为患者提供了除标准化疗以外更多的治疗选择。但是，生物标志物PD-L1的表达在预测ICIs治疗MPM疗效中的作用有限。鉴于ICIs治疗仅可为部分患者带来长期生存获益，需要进一步研究和探索可以预测治疗效果的生物标志物，以期实现个体化精准治疗。
